# Androgen receptor signaling and spatial chromatin organization in castration-resistant prostate cancer

**DOI:** 10.3389/fmed.2022.924087

**Published:** 2022-07-29

**Authors:** Tianyi Zhou, Qin Feng

**Affiliations:** Center for Nuclear Receptors and Cell Signaling, Department of Biology and Biochemistry, University of Houston, Houston, TX, United States

**Keywords:** androgen, steroid hormone receptor, castration resistant, prostate cancer, phase separation, TAD

## Abstract

Prostate cancer is one of the leading causes of cancer death and affects millions of men in the world. The American Cancer Society estimated about 34,500 deaths from prostate cancer in the United States in year 2022. The Androgen receptor (AR) signaling is a major pathway that sustains local and metastatic prostate tumor growth. Androgen-deprivation therapy (ADT) is the standard of care for metastatic prostate cancer patient and can suppress the tumor growth for a median of 2–3 years. Unfortunately, the malignancy inevitably progresses to castration-resistant prostate cancer (CRPC) which is more aggressive and no longer responsive to ADT. Surprisingly, for most of the CPRC patients, cancer growth still depends on androgen receptor signaling. Accumulating evidence suggests that CRPC cells have rewired their transcriptional program to retain AR signaling in the absence of androgens. Besides AR, other transcription factors also contribute to the resistance mechanism through multiple pathways including enhancing AR signaling pathway and activating other complementary signaling pathways for the favor of AR downstream genes expression. More recent studies have shown the role of transcription factors in reconfiguring chromatin 3D structure and regulating topologically associating domains (TADs). Pioneer factors, transcription factors and coactivators form liquid-liquid phase separation compartment that can modulate transcriptional events along with configuring TADs. The role of AR and other transcription factors on chromatin structure change and formation of condensate compartment in prostate cancer cells has only been recently investigated and appreciated. This review intends to provide an overview of transcription factors that contribute to AR signaling through activation of gene expression, governing 3D chromatin structure and establishing phase to phase separation. A more detailed understanding of the spatial role of transcription factors in CRPC might provide novel therapeutic targets for the treatment of CRPC.

## Introduction

Prostate cancer is one of the most frequently diagnosed cancers in America. It is the second most common cause of cancer death in American men. During the progression of prostate cancer, androgen receptor (AR) functions as a critical regulator that governs cancer development through transcriptional regulation of its targets gene expression (1). Androgen deprivation therapy (ADT) has been developed to inhibit AR signaling and has been considered as the golden standard in treating prostate cancer (2). The disease can be controlled for a certain period of time, but the tumors typically recur after an average of 2–3 years of ADT treatment (3). The recurrent tumors are usually more aggressive and insensitive to additional ADT treatment (4). These tumors are known as castration-resistant prostate cancer (CRPC) (3, 5). CRPC is currently incurable and can further progress to deadly metastatic prostate cancer. There is an urgent need to develop novel effective therapeutic strategies for CRPC.

There is growing evidence that CRPC cells have rewired their transcriptional program to recover and maintain AR signaling in the absence of androgens. The alteration of transcription factors and their associated chromatin modifiers contribute to the resistance mechanism through multiple pathways. These changes also shape the higher order of chromatin structure, affect gene expression, and consequently allow the progression of CRPC and therapy resistance. This review is focused on androgen receptor, AR-related transcription factors, and their associated spatial organization of genome in CRPC. A more comprehensive review on clinical and molecular alterations during development of CRPC has been summarized in another review (6).

## Androgen receptor

The Androgen receptor (AR) is the receptor protein mediating the androgen action and essential for prostate cancer development. It is encoded by the AR gene located on X chromosome with eight exons. AR contains three distinct functional domains and one hinge region. The functional domains include an NH_2_-terminal transcription activation domain (NTD), the central DNA binding domain (DBD), and a carboxyl-terminal ligand binding domain (LBD). The AR DBD specifically recognizes the androgen response elements (ARE) that contain a palindromic dihexameric motif with 5′-AGAACA-3′ core sequence (7). DBD is also the most conserved domain that contains two zinc finger polypeptides (8). The binding of AREs by DBD allows androgen receptor to regulate its target genes specifically. Binding of the AR LBD with androgens in the cytoplasm exposes the nuclear localization signal (NLS) and results in the nuclear entry of AR.

By classical transient transfection/luciferase-based transcriptional assays, two transactivation regions were identified in AR, namely Activation Function 1 (AF1) and Activation Function 2 (AF2). The ligand-independent AF1 is located in the AR NTD, and the ligand-dependent AF2 is located in the AR LBD. In contrast to many other nuclear receptors that AF2 regions are responsible for the dominant transactivation activity, the AR AF1, rather than AF2, plays a major role in determining the transactivation activity of AR (9, 10). This observation was supported by the recently solved cryo-EM (electron cryo-microscopy) structure of full-length AR and coactivator complex. It was directly visualized that the AR AF1 is the primary site for recruitment of transcriptional coactivators such as p300 and SRC-3 (11).

Androgen receptor can be activated by its natural agonists testosterone and dihydrotestosterone and is the major target for prostate cancer therapy (12, 13). Early-stage prostate cancer can be treated by androgen deprivation therapy to lower the levels of androgens or by antiandrogens, which compete with androgens for binding to the LBD of AR. As a result, these treatments disrupt the AR signaling (14). Additionally, certain antiandrogens can alter the conformation of AR and further impair its transactivation activity. For instance, the antiandrogen enzalutamide not only blocked the binding of androgens to AR, but also prevented the nuclear entry of AR and the exposure of AR DBD (15). Consequently, enzalutamide-bound AR loses the ability to bind to AREs on chromatin. However, clinical studies showed that majority of patients eventually developed resistance to enzalutamide typically within a year (16–19). Although it was initially believed that these resistant tumors are no longer dependent on the AR signaling (20), later studies have demonstrated that majority of CRPC are still dependent on AR and the AR signaling (21–24).

## Molecular alterations of androgen receptor in castration-resistant prostate cancer

**FIGURE 1:**
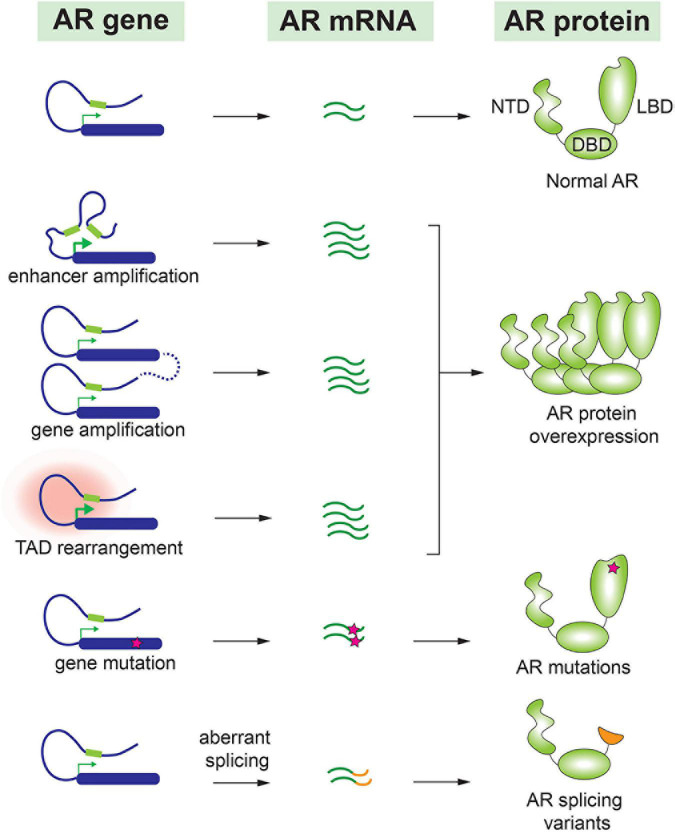
Molecular alteration of AR in CRPC.

To adapt to the minimal levels of androgens after castration, malignant cells have undergone various genetic alterations to preserve the AR signaling pathway. As a central player for the AR signaling, AR gene expression level is frequently augmented in CRPC. This can be achieved by several molecular alterations, including amplification of AR gene and a distant enhancer, change of chromatin interactions, translational regulation of AR mRNA, and increase of AR protein stability. AR gene amplification and copy number alterations are detected in up to 60% of metastatic CRPC patients but not in untreated primary prostate tumors (25–30). Matthew Meyerson and his colleagues performed linked-read whole genome sequencing analysis on 23 biopsy specimens from metastatic CRPC, and they observed complex rearrangements of the AR locus in most cases. 78–87% of these CRPC samples contained tandem duplications involving an upstream enhancer of AR, indicating that structural alterations in the non-coding genome largely contribute to sustained AR signaling in CRPC (31). David Takeda and his colleagues identified a vestigial enhancer approximately 650 kb centromeric to AR gene (32). This region was frequently amplified in CRPC tumors, but the amplification was rarely seen in primary localized tumors. The common features of enhancers, such as acetylation of histone H3K27 and clustering of multiple transcription factor binding sites, were associated with this enhancer in CRPC cells. Most interestingly, genomic knock-in of this enhancer region increased the expression level of AR and conferred a castration-resistant state to prostate cancer cells (32). Additionally, ChIA-PET of RNA Pol II in prostate cancer cells revealed that the amplified AR locus further increases the number of chromatin interaction modules containing AR gene and its distal enhancer, which maximizes the upregulation of AR gene expression (33). Therefore, by increasing AR expression to compensate for the reduced androgens is a common mechanism to preserve the AR signaling in CRPC (Figure 1).

Accumulating evidence indicates that AR splice variants are implicated in the drug resistance of CRPC (34–36). Most AR splice variants lack LBD, and among all AR variants, AR-V7 and AR-V567es are most studied. Truncation of LBD enables these splice variants to be constitutively active even in the absence of androgens (37). AR-V7 has been detected in many CRPC patients (28, 38) and is considered as a major cause of resistance to antiandrogen drugs such as enzalutamide and abiraterone (39–41). In fact, the prevalence of AR-V7 is 9–15% in patients who hadn’t taken any antiandrogen drug, in contrast to approximately 50% in patients who underwent enzalutamide or abiraterone treatment (39). Although some studies implied that AR-V7 was able to recover partial transcriptional activity of full-length AR under the castration condition, others reported that AR-V7 gained new functions to drive the progression of CRPC (42, 43). For instance, AR-V7 was observed to interact with full length AR and consequently recruited transcriptional corepressors rather than coactivators. This repression mechanism led to downregulation of a subset of growth-suppressive genes therefore contributing to the development of CRPC (44). Similarly, AR-V567es functioned as a constitutively active receptor, and it formed heterodimers with full-length AR and increased the overall transcriptional activity of AR (45). In transgenic mouse model with prostate gland-specific expression of AR-V567es, epithelial hyperplasia was observed in 4 months and invasive adenocarcinoma were developed in 12 months, indicating that AR-V567es promotes prostate tumorigenesis *in vivo* (46). This splice variant was detected in 23% of CRPC bone metastases and was associated with poor survival (35).

In addition to AR splice variants, AR point mutations have been frequently detected in CRPC but rarely seen in untreated prostate tumors (26, 28). Interestingly, most of these point mutations are located in the LBD of AR. Earlier work reported that mutated AR acquired the ability to be activated by other steroid hormones such as glucocorticoid and progesterone (47, 48). More recent publications have confirmed these observations. For instance, a frequently detected mutation, AR-L702H, enables the receptor to be activated by prednisolone and cortisol (49). In addition, AR-F877L mutant could even convert the action of an antagonist to become an agonist (50). Therefore, it was not surprising that these AR point mutations were associated with resistance to the second-generation antiandrogen drugs (51, 52).

## Steroid hormone receptors in castration-resistant prostate cancer

Androgen receptor belongs to the nuclear receptor superfamily, which contain less conserved NH2-terminal transactivation domain and more conserved DBD and LBD (53). Within this superfamily, AR and four other members including estrogen receptor (ER), glucocorticoid receptor (GR), progesterone receptor (PR), and mineralocorticoid receptor (MR) are more evolutionarily conserved and functionally related. These receptors are also known as steroid hormone receptors. The structures of their LBDs, their cognate ligands, and the DNA sequences of their response elements are highly homologous, and these receptors directly transmit the signal of environmental steroid hormones to nuclear gene expression to regulate cell proliferation, metabolism, immunity, and sexual development. In CRPC, cancer cells have taken advantage of these steroid receptors to establish drug resistance.

Glucocorticoid receptor was found upregulated in a subset of CRPC and partially substituted for the AR to activate gene expression. The GR DBD shares more than 70% homology with the AR DBD, and thus it can recognize the same response element as the AR (54). In enzalutamide resistant CRPC, upregulated GR was found to bind to ARE and activated a subset of AR target genes. Dexamethasone, a GR agonist, was able to confer enzalutamide resistance, and in CRPC patients, higher expression levels of GR were correlated with poor response rate to enzalutamide treatment (55, 56). These studies suggested that GR might be a therapeutic target for progressive CRPC. However, a recent clinical trial of combination therapy with enzalutamide and GR antagonist mifepristone neither delayed PSA progression, nor prolong radiographic or clinical progression-free survival of the patients (57).

There are two estrogen receptors (ERs), ERα and ERβ, that are encoded by different genes on chromosome 6 and 14, respectively. They share similar protein structure but ERβ is the prevalent ER expressed in human prostate tissue (58). It is generally considered that ERα has tumor-promoting activity whereas ERβ behaves as a tumor suppressor through antagonizing ERα and inhibiting cell proliferative pathways (59). The ERα expression was elevated in CRPC, accompanied by increased expression of its target genes. A clinical trial with rapid androgen deprivation also induced upregulation of ERα expression in malignant epithelia, which was correlated with cell proliferation (60). In contrast to the ERα, ERβ expression starts to decline at early stage of prostate cancer development and further decreases in CRPC. This might be due to ERβ being an AR target gene (61). On the other hand, ERβ down-regulates AR in a negative feedback loop in prostate cancer cells. A study from Gustafsson’s group suggested that ERβ could suppress cancer cell proliferation by repressing AR signaling. Agonist-activated ERβ limited expression of AR downstream gene such as CaMKK2, which was thought to be one of the key regulatory factors during the establishment of castration resistance (62–65).

MR is ubiquitously expressed in various tissues. The expression of MR is low in prostate cancer cells, but studies have showed that MR might be also involved in enzalutamide resistance in CRPC (66, 67). Mineralocorticoid and glucocorticoid can both bind to the LBD of MR and exert different biological functions. A group at Kyushu University performed a clinical study in which 86 patients were administrated with enzalutamide and corticosteroids. Co-treatment of MR agonist aldosterone increased the sensitivity to enzalutamide whereas knockdown of MR promoted enzalutamide resistance and AR signaling (67). Although the underlying mechanism was not completely understood, this study suggested that crosstalk of steroid hormone receptors, such as AR and MR, existed in CRPC. When androgen signaling is diminished under ADT, other steroid receptors may substitute and partially compensate for the transcriptional activity of AR. Collectively, the steroid receptor ligand-binding versatility and DNA-binding promiscuity may be one underlying mechanism of castration resistance (68).

## Topologically associating domains (TADs)

Human genome is organized based on spatial proximity into different architectural chromatin units known as the topologically associating domains (TADs). Within TADs, the embedded genes can share similar transcription factors and coregulators. Different gene elements, including enhancers, promoters, or gene bodies, have more frequent interactions with each other. TADs are separated by TAD boundaries, which are the regions enriched with insulator proteins, such as the CCCTC-biding factor (CTCF) protein. As it was named, CTCF binds to the core sequence CCCTC and was first identified as a transcriptional repressor of chicken c-myc gene (69). Later CTCF was found to be an insulator blocking transcriptional activation when located between enhancer and promoter (70, 71). More recent studies have established its causal role in TAD formation and maintenance (72–74). The organization of the TADs are tightly associated with the control of gene transcription. Disruption of TAD boundaries may result in aberrant activation of genes both in cultured cells and *in vivo* (75, 76).

In 2016, Susan Clark and her colleagues (77) reported the first comparative analysis of chromatin organization between cancerous and normal prostate cells. Intriguingly, the overall spatial organizations of chromatin were quite similar in these cells, but the size of individual TADs appeared smaller in cancer cells. New cancer-specific chromatin interactions were detected within these smaller TADs (77). Indeed, in prostate cancer cells, the boundaries established by CTCF showed more effective AR transcriptional regulation than in normal cells (78). Similarly, in another study, genomic alterations of 23 metastatic CRPC tumors were analyzed by linked-read whole genomic sequencing; tandem repeats of AR enhancer element were identified, but the alteration appeared to be limited within the same TAD (31). These studies suggested that the spatial organization of certain TADs are stable during the prostate tumorigenesis. In a follow-up study, the effect of CTCF on the organization of TAD was further explored in prostate cancer cells. When CTCF was knockdown by RNAi, the overall level of CTCF protein was markedly reduced. Interestingly, a subset of CTCF-bound sites remained intact. Deletion of two of these persistent CTCF sites by CRISPR-Cas9 genome editing resulted in aberrant expression of a few genes in proximity to the CTCF binding sites, indicating that these persistent CTCF binding sites were essential in maintaining the cell-type constitutive, higher order chromatin architecture (79).

**FIGURE 2:**
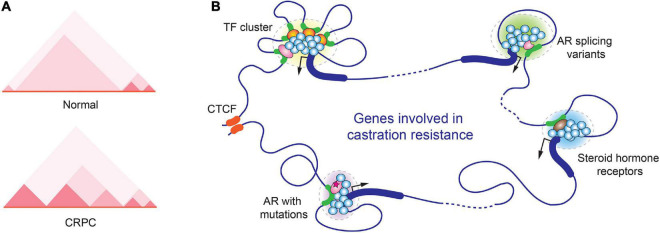
Alterations in spatial organization of chromatin in CRPC. **(A)** Chromatin structure changes in the genomic region near the AR gene locus in normal prostate and CRPC cells. A representative TAD heatmap is illustrated. **(B)** A hypothetic schematic model for various transcription-associated condensates formed in CRPC.

Later on, Peggy Farnham’s group performed *in situ* Hi-C and ChIP-seq experiments in prostate cancer cells to determine how three-dimensional chromatin structure is correlated to prostate cancer transcriptome. This study generated chromatin interaction map in high resolution and identified more than a thousand of TADs with altered boundaries in prostate cancer cells (80). In CRPC cells 22Rv1, cancer cell-specific new smaller TADs were identified at AR gene locus, and this alteration was believed to contribute to the exotic expression of AR-V7 in 22Rv1 (Figure 2A) (80). Moreover, based on GWAS studies, the same group identified ∼300 prostate cancer risk-associated SNPs that were enriched in active regulatory regions, including promoters, enhancers, insulators, and chromatin loop anchors. Deletion of two prostate cancer risk-associated CTCF anchor regions by CRISP-Cas9 resulted in highly elevated expression of genes within the loops, suggesting that disruption of TAD organization could alter gene expression and contribute to prostate tumorigenesis (81).

Recently, Rachel Patton McCord’s group analyzed the genome organization of nine cell lines that mimic different stages of prostate cancer progression by Hi-C. CRPC cell lines 22Rv1 and C4-2B were included in the study. Across prostate cancer progression, 386 genes were found switching from transcriptionally silenced compartments to transcriptionally active compartments along with TAD rearrangement. In addition, they further defined several “genomic architecture hotspots,” where the chromatin alterations were highly similar among all metastatic models. Some important genes in prostate tumorigenesis, including AR, WNT5A, and CDK14, were located in these hotspots. This study suggested that the changes of 3D genome structure are critical for prostate cancer progression (82).

## Phase separation

Liquid-liquid phase separation (LLPS) represents a dynamic phenomenon that two distinct liquid phases are demixed (83). LLPS is a common process that utilized by our cells to accomplish complex biochemical reactions and to respond to cellular stress (84, 85). Many membraneless subcellular structures are organized as LLPS, also known as condensates, bodies, granules, or droplets, depending on their appearance under microscope. Well studied LLPS structures include stress granules and P bodies in the cytoplasm, nucleoli, Cajal bodies, paraspeckles, promyelocytic leukemia (PML) bodies and histone locus body (HLB) in the nucleus (86–90). It is generally accepted that biomolecules are highly enriched inside LLPS, allowing a condensed spatial distribution of biomolecules and therefore more efficient biochemical reactions (91).

Similar to many other biological processes, gene expression also takes advantage of LLPS to maximize the output of gene transcription. To achieve high density of biomolecules in LLPS, transcription factor binding sites frequently form clusters on gene regulatory regions including enhancers and promoters. In AR-regulated genes, the response elements of pioneer transcription factors of AR, such as Forkhead Box A1 (FOXA1) and GATA-binding protein 2 (GATA2), were often found in close proximity to ARE (92–94). The multivalent interactions between transcription factors and coregulators stabilize the transcriptional initiation complexes and contribute to LLPS formation during transcriptional initiation (95–97). Steroid hormone receptors in CRPC, including AR, GR, ER, all have been reported to induce transcription through phase separation (98–101). In one study using GFP-tagged AR to examine its cellular localization in response to agonists and antagonists, it was found that AR localized to nuclear foci in the presence of the agonist R1881 but not the antagonist bicalutamide and hydroxyflutamide. However, the CRPC mutants AR-W741C and AR-T877A regained the ability to form the nuclear foci (102). The study suggested that the LLPS formation and transcriptional activation are coupled events (Figure 2B).

One major characteristic of proteins involved in LLPS is that they usually contain intrinsically disordered region (IDR). IDR refers to a protein region that does not form a fixed three-dimensional structure, but rather adopts flexible conformation (103). The dynamic interactions between various IDRs help to enrich transcriptional regulators to form transcription-related condensates (104, 105). Because the NH_2_-terminal domains of steroid hormone receptors all contain IDRs, it is conceivable that these NTDs play a role in forming phase separation (99, 106). Indeed, the recombinant NTD of AR was able to form LLPS *in vitro*. Within the NTD, the low complexity poly Q sequence appeared to be essential for phase separation (107). Interestingly, AR DBD could also form LLPS through binding with RNA and DNA molecules (108). Although these AR functional domains were able to form liquid condensates as high concentrated recombinant proteins *in vitro*, it is unknown if such level of AR concentration could be achieved inside the cells, even within the transcription-associated condensates. It is likely that additional biomolecules, such as transcription factors, transcriptional coactivators, enhancer RNA molecules, and phosphorylated RNA polymerase II, are involved to ensure the establishment of LLPS and a robust transcription to occur (106, 109).

A very recent study showed that AR formed transcriptionally active condensates with coactivator MED1 in androgen-responsive prostate cancer cells such as VCaP and LAPC4 cells, but not in RWPE1, a normal prostate epithelial cell line. Interestingly, these condensates were only observed with full-length AR upon DHT stimulation, but not with truncated AR mutants such as AR-V7 (110). This study indicates that formation of LLPS is not only determined by its protein composition, but also affected by cellular context and various cell signals. Another study in CRPC identified Octamer-binding transcription factor 4 (OCT4) as a key molecule to collaborate with other transcription factors on super enhancers and promoters to drive cancer cell proliferation and castration-resistance. In AR-positive prostate cancer cells, OCT4 forms LLPS with the AR and pioneer transcription factor FOXA1. In AR-negative prostate cancer cells, OCT4 forms LLPS with Nuclear respiratory factor 1 (NRF1). This study suggests that targeting collaborations between transcription factors might be a novel therapeutic strategy for CRPC treatment (111).

Aside from being involved in gene transcription, LLPS is implicated in other regulatory pathways in prostate cancer. In a subset of advanced CRPC where the AR protein level was low or absent, DEAD-box helicase 3 X-linked (DDX3) was reported to directly repress AR protein translation by physical association with AR mRNA in cytoplasmic liquid condensates. The upregulated DDX3 might contribute to the castration resistance in these tumors (112). Moreover, several studies on Speckle-type POZ protein (SPOP) further expanded our understanding on the role of LLPS in prostate carcinogenesis. SPOP is a substrate adaptor of the cullin3-RING ubiquitin ligase, and SPOP mutations have been frequently detected in prostate tumors (113–115). SPOP formed nuclear condensates with its substrates, but its cancer-associated mutations disrupted their colocalization and liquid phase separation, resulting in impaired substrate ubiquitination and accumulation of oncogenic proteins such as c-MYC, SRC-3, and DAXX (113, 116–118). Although how these biomolecules regulate LLPS condensates in CRPC remain poorly characterized, these pioneer studies support the importance of LLPS in tumor progression and development of drug resistance.

## Connection between topologically associating domains and liquid-liquid phase separation

Studies have shown that CTCF not only functions as an insulator to establish the topological structure of TADs, but also is involved in sub-TAD chromatin loop formation within the TADs (78, 119–121). In prostate cancer, the expression levels of CTCF showed strong association with the levels of proliferation marker Ki67, advanced pathological tumor stage, nodal metastasis, and early biochemical recurrence (122). It remains unknown if CTCF level is associated with organization of transcriptional condensates in prostate cancer cells, but studies in colorectal cancer cells showed a positive correlation. Depletion of CTCF altered the genome-wide TAD insulation and resulted in disruption of phase separated transcriptional condensates in colorectal cancer cells, indicating that CTCF-mediated DNA looping provides a spatial architecture and essential for the formation of transcriptional condensates (123).

Contrariwise, it appears that LLPS could also influence TAD assembly. One study applied a new CRISPR-Cas9-based optogenetic technology named CasDrop, which could induce microscope-visible liquid condensates to form at specific genomic loci. It was found that nuclear condensates, while they were forming, were able to gather the distal targeted genomic elements and expand to euchromatic regions (124). Another study showed that during the ER and PR-mediated transcriptional activation, the assembled transcriptional complexes reorganized the looping structures in TADs and fine-tune the three-dimensional genome folding (125). It remains to be determined if LLPS alteration affects organization of TADs or chromatin looping in CRPC.

## Future perspective

With the advancement of next generation genome analysis technologies such as Hi-C, DNase-seq, FAIRE-seq, and ATAC-seq, we have learned that when hormone-sensitive prostate cancer progresses to castration-resistant prostate cancer, many genomic alterations at chromatin level have occurred. These genomic alternations underlie transcriptional reprogramming during the establishment of castration resistance. Recent advances in Hi-C and super resolution microscopes allow researchers to further characterize the chromatin conformation and transcriptional condensates in a spatial and dynamic view. The studies discussed in this review represent early steps toward understanding how the higher order of genomic organization affects the development of CRPC, yet more questions need to be addressed in future studies. It is hoped that these mechanistic studies could promote the development of new therapeutic interventions for the treatment of CRPC, a currently incurable disease.

## Author contributions

TZ and QF conceived and wrote the manuscript. Both authors contributed to the article and approved the submitted version.
